# Association between maxillary canine impaction and other dental anomalies: radiological study of a mixed dentition children’s cohort from an orthodontic clinic

**DOI:** 10.1007/s40368-023-00798-y

**Published:** 2023-04-13

**Authors:** O.-E. Kolokitha, D. Balli, A.-E. Zarkadi, S. Gizani

**Affiliations:** 1 Department of Orthodontics, Faculty of Dentistry, School of Health Sciences, Aristotle University of Thessaloniki, Thessaloniki, Greece; 2 Department of Pediatric Dentistry, Faculty of Dentistry, School of Health Sciences, Aristotle University of Thessaloniki, Thessaloniki, Greece; 3 Head of Paediatric Dentistry, Department of Dentistry, Kapodistrian University of Athens (NKUA), Athens, Greece

**Keywords:** Tooth anomalies, Maxillary canine, Canine impaction, Orthodontic subjects

## Abstract

**Purpose:**

To investigate the significance of association between maxillary impacted canines and various dental anomalies.

**Methods:**

Files of 874 orthodontic patients were evaluated for the presence of maxillary impacted canines. From this sample, a group of 97 patients (39 males and 58 females) with at least 1 impacted maxillary canine consisted the study group. This group was compared to a control group of 97 patients (42 males and 55 females) that was created by random selection from the initial sample without maxillary canine impaction. The impaction diagnosis was made from the panoramic radiographs. Chi-square test was used to perform the analysis for significant associations. Stepwise discriminant analysis, binary logistic regression and classification tree were used to identify best combinations.

**Results:**

Statistically significant difference was found for peg-shaped maxillary lateral incisors and infraoccluded deciduous molars. The presence of peg-shaped upper lateral incisors arises the probability of impacted canine to 83.3%, a distal displaced unerupted second premolar to 63.16% and the impaction of any other teeth to 80% as showed by the classification tree.

**Conclusions:**

The presence of peg-shaped maxillary lateral incisors and infraocclusion of deciduous molars can be considered major valuable early risk indicators for maxillary canine impaction, because they manifest before the maxillary canine eruption. Special consideration should be given on distal displaced unerupted second premolar and the impaction of any other teeth. Patients with these dental anomalies are candidates for future interceptive treatment for canine eruption.

## Introduction

Impaction of permanent maxillary canine is a developmental disturbance of eruption which is important to dentistry and more important in orthodontics. Maxillary canines are the second most frequent impacted teeth after third molars with a prevalence fluctuating from 1 to 6% (Lövgren et al. 2019; Sambataro et al. 2004; Afify et al. 2012; Herrera-Atoche et al. 2017).

Several studies aimed to identify etiological factors or causative genes that are involved in the mechanism of the impaction and eruption of maxillary canines. It has been identifying that long path of eruption, dilaceration of the root, lack of space, trauma, the presence of an alveolar cleft, a cystic lesion could be etiological factors for maxillary canine impaction. (Peck et al. 1994; Stellzig et al. 1994; Becker and Chausu 2015; Russell and McLeod 2008). Regrettably, the underlying mechanisms involved in the impaction of the maxillary canines have been not clarified yet. Two theories related to the etiology of maxillary canine impaction have been proposed. The guidance theory suggests that in case of absence or malformation of the root or the crown of the incisor, the canine has no guidance for its eruption, therefore, it remains impacted. Insufficient or absent root of the lateral incisor is not capable of creating the proper direction for the eruption pathway of the canine. (Becker et al. 1981, 2002, 1999; Kim et al. 2017). The genetic theory relates the impaction of canines with genetic factors (Peck et al. 1994; Baccetti 1998; Al-Nimri and Bsoul 2011; Devi and Padmanabhan 2019; Vitria et al. 2019). The increased frequency of impacted canines among family members and the linkage of impacted canines with a variety of other genetically controlled dental anomalies are the main content of this theory (Stahl et al. 2003; Baccetti 1998). Peck et al (1994) found a genetic background of palatal displaced canines which was based on different prevalence between ethical races and genders, increased prevalence in the same family and of other dental anomalies. Peck et al (1996) substantiated a common genetic control between hypodontia, tooth-sized reduction and canine impaction. In 2002, the same research team added maxillary canine-first premolar transposition in the category of dental anomalies that are strongly connected with canine impaction. According to Baccetti et al (2010), distally displaced mandibular second premolars are also related with impacted canines. Singer et al. (2011) showed that there is association between small lateral incisors, infraocclusion of deciduous molars, distoangulation of mandibular second premolar and impacted maxillary canines.

Tooth impactions, supernumerary teeth, oligodontia, infraoccluded teeth, taurodontism and ectopic eruption of mandibular canines are examples of dental anomalies presented in a not negligible portion of people around the world and constitute a team of dental manifestations frequently examined in patients with maxillary impacted canines (Patil et al. 2013; Lagana et al. 2017; Shokri et al. 2014; Lempesi et al. 2014; Pallikaraki et al. 2019). These dental anomalies could work as risk indicators of maxillary canine impaction and contribute to their early diagnosis and treatment, since they are detected earlier in the oral cavity (Napgal et al. 2009; Leifert et al. 2003; Becker et al. 1981; Shalish et al. 2010; Baccetti et al. 2010; Herrera-Atoche et al. 2017; Garib et al. 2017).

Complex, prolonged, difficult, and precarious are only some of the adjectives used in scientific literature to describe the treatment of impacted canines. Additionally, the possibility of external root resorption of adjacent teeth (Ericson and Kurol 2000; Woloshyn et al. 1994), crest bone loss at the mesial aspect of the canine (Bishara 1992) and the uncertainty of maintaining a good long-term periodontal health of the tooth (Parkin et al. 2013) are some consequences of canine impaction treatment. When diagnosed at an early age, simple preventive treatment approaches such as deciduous canine extraction (Ericson and Kurol. 1988), cervical traction (Baccetti et al. 2008), rapid maxillary expansion (Baccetti et al. 2009) or their combination (Sigler et al. 2011) may lead to spontaneous canine eruption (Garib et al. 2016). Therefore, the recognition of risk indicators could be very helpful for early diagnosis and preventive treatment of impaction. The aim of the present study is to evaluate the significance of association between maxillary canine impaction and other dental anomalies.

## Methods

An initial sample of 874 orthodontic patients at the mixed dentition, from the files of the same private orthodontic practice were examined. All patients had a complete pretreatment record. From this sample, a group of patients with at least one impacted maxillary canine were selected. The impaction diagnosis was made from the panoramic radiographs and confirmed during surgery. Ninety-seven patients (39 males and 58 females) with a mean age of 10.3 ± 1.2 years were found with at least 1 maxillary impacted canine and consisted the study group (impacted canines) of this research. Inclusion criteria for enrollment in this study were: Caucasian race, no previous orthodontic treatment and good quality of panoramic radiographs. Exclusion criteria were craniofacial syndromes and history of trauma. The study group was compared with a control group. Control group consisted of 97 subjects (42 males and 55 females) with a mean age of 10.5 ± 1.2 years without maxillary canine impaction that had been randomly selected from the initial sample.

Study group and control group were examined for dental anomalies. The panoramic radiographs were evaluated on the computer screen for the potential dental anomalies by three observers. The evaluation was performed in a darkened room to enhance the visibility of the radiographs. The following anomalies were detected by direct observation:

1. Agenesis of any permanent tooth (except third molars). Maxillary lateral incisor agenesis and mandibular second premolar agenesis were evaluated separately. Agenesis is defined as the inherent absence of one or more teeth in primary or permanent dentition (Qamar et al. 2012).
2. Maxillary lateral incisor agenesis.
3. Mandibular second premolar agenesis.
4. Peg-shaped maxillary lateral incisors. Defined as small maxillary lateral incisor with severe size reduction of the crown, in some cases associated with narrowing in diameter from incisal edge to the cervix (Baccetti 1998).
5. Infraocclusion of premolar. Considered in infraocclusion when it was positioned below the occlusal plane in a variety of vertical discrepancies.
6. Infraocclusion of primary molars. Is considered when the occlusal plane of the primary molar is apically positioned relative to the occlusal plane of the adjacent teeth (Sigler et al. 2011).
7. Distal displacement of mandibular second premolar. Defined as a distal displacement of unerupted mandibular second premolar.
8. Tooth transpositions. Defined as positional interchange of the two adjacent teeth—particularly of the roots—or the development or eruption of a tooth in a position occupied normally by a non-adjacent tooth (Peck et al. 1993).
9. Impaction of other teeth (maxillary and mandibular third molars were excluded because of the more delayed eruption of these teeth). Impacted tooth defined as a tooth that is prevented from erupting into position because of malposition, lack of space, or other impediments, or as a tooth that fails to erupt into the dental arch within the expected time, or as o tooth prevented from eruption due to a physical barrier within the path of eruption (Peterson 1998; Agarwal et al. 2004).
10. Supernumerary teeth. Defined as one tooth that is additional to the normal series and can be found in almost any region of the dental arch (Garvey et al. 1999).
11. Taurodontism. Considered when the tooth body and/or pulp chamber is enlarged vertically, and the pulp chamber is in a rectangular configuration (Dineshshankar et al. 2014).
12. Dens evaginatus. A developmental anomaly characterized by the presence of an accessory cusp-like structure projecting from the cingulum area or cementum-enamel junction of the maxillary or mandibular anterior teeth in both the primary and permanent dentition (Hattab et al. 1995).
13. Dens invaginatus. A developmental anomaly characterized by an enfolding in the mineralized portion of the tooth (Rotstein et al. 1987).
14. Fusion. Recognized as a union of two separate tooth buds at some stage in their development with confluence of dentin; pulp chambers and canals may be joined or separate, depending on the amount of development at the time. There is one tooth fewer than the normal count for the arch if the affected tooth is counted as one (Duncan and Helpin 1987).
15. Germination. Recognized as an attempt by a single tooth germ to divide, with a resultant large single tooth with bifid crown and usually a common root and root canal. The normal number of teeth is found, if the affected tooth is counted as one (Duncan and Helpin 1987).
16. Ectopic eruption of mandibular canine. Tooth eruption is the process whereby the newly formed tooth moves from its internal location to its functional position within the oral cavity (Vijayendranath et al. 2017).
17. Μesial rotation of mandibular canine.

### Statistical analysis

The frequencies for all dental anomalies detected in this study were measured. Study group was compared with control group for every anomaly. Chi-square test was used to perform the analysis for significant associations. Results considered to be significant when the *p*-value was lower or equal of 0.05. Stepwise discriminant analysis and binary logistic regression were used to identify best combinations of dental anomalies and impacted canines. Apart from the mathematical parametric models, an attempt to find combinations with a classification tree was made.

The reproducibility of diagnoses was assessed by re-examining the records of 25 patients 2 weeks after the first examination. Reproducibility was 100% for all variables.

## Results

From the initial sample of 874 patients, 97 patients with a mean age of 10.3 ± 1.2 years were found with at least 1 impacted maxillary canine. From these, 39 were males and 58 females. With respect to gender distribution, impaction group consisted of 40.2% males and 59.8% females.

Table 1 reports the prevalence and distribution of every dental anomaly (variable) examined and a *p*-value (significance) of a chi-square test showing the association of each variable with the control/impacted group or, in other words, the difference between control and impacted groups with respect to the specific variable. The level of significance is 0.05, so a value of *p* < 0.05 shows significant association.

**Table 1:** Distribution of variables with respect to control/study group

	Study	Group	Control	Group	*P*-value
Variables	Patients	%	Patients	%	
Agenesis of any permanent teeth, except for third molars	8	8.2	9	9.3	0.800
Maxillary lateral incisor agenesis	9	9.3	4	4.1	0.151
Mandibular second premolar agenesis	4	4.1	4	4.1	1.000
Cone-shaped upper lateral incisors	15	15.5	3	3.1	**0.003**
Infraocclusion of premolars	0	0.0	1	1.0	0.316
Infraocclusion of deciduous molars	5	5.2	0	0.0	**0.023**
Distoangulation of mandibular second premolars	12	12.4	7	7.2	0.227
Tooth transposition	3	3.1	1	1.0	0.312
Impaction of other teeth	4	4.1	1	1.0	0.174
Supernumerary teeth	0	0.0	1	1.0	0.136
Ectopic eruption of mandibular canine	15	15.5	8	8.2	0.120
Mesial rotation of mandibular canine	25	25.8	24	24.7	0.869

*P* < 0.05 shows significant association

Increased prevalence rate was found for distal displacement of unerupted mandibular second premolar (12.4% in the impaction group and 7.2% in the control group) and ectopic eruption of mandibular canine (15.5% in the impaction group and in control group 8%). The prevalence rate of maxillary lateral incisor agenesis was 9.3% for the impaction group and 4.1% for the control group. Supernumerary teeth and infraocclusion of premolars were not frequent findings in any group. In contrary, mesial rotation of mandibular canine was a frequent finding in both groups. The prevalence rate for mandibular second premolar agenesis was found the same for both groups.

Dens invaginatus, dens evaginatus, taurodontism, germination, fusion and anomalous mandibular canine not found in any group. For that reason, they were not considered as actually variables and do not appear as those in tables.

Statistically significant difference was found for peg-shaped maxillary lateral incisors and infraoccluded primary molars. The impaction group exhibited significantly greater prevalence of peg-shaped lateral incisors (*p* = 0.003 < 0.05) and infraoccluded primary molars (*p* = 0.023 < 0.05) than the control group. The prevalence rate of peg-shaped lateral incisors in the impacted group is 15.5%, while in the control group, only 3.1%. The corresponding percentage for infraoccluded molars is 5.2% and 0%, respectively.

There were attempts to find some complex relations between the variables with respect to the impaction/control groups. Stepwise discriminant analysis and binary logistic regression confirm the above result that peg-shaped upper lateral incisors and infraocclusion of primary molars are the most important variables for the discrimination between control and impacted groups. The sex of the subjects was included in the Stepwise discriminant analysis, but no effect was found.

Apart from the mathematical parametric models, an attempt to find relations with a classification tree was made. The results are seen at Table 2. The values of all variables are represented by 0 = No, 1 = Yes, so < 0.5 means No and > 0.5 means Yes. The presence of a variable (dental anomaly) represents the “Yes” category and the absence of a variable the “No” category. For the “Yes” category of the variable, peg-shaped upper lateral incisors the probability of being in the impacted group is 83.3%. This variable is found the most significant for the classification of impacted/control group.

**Table 2:**
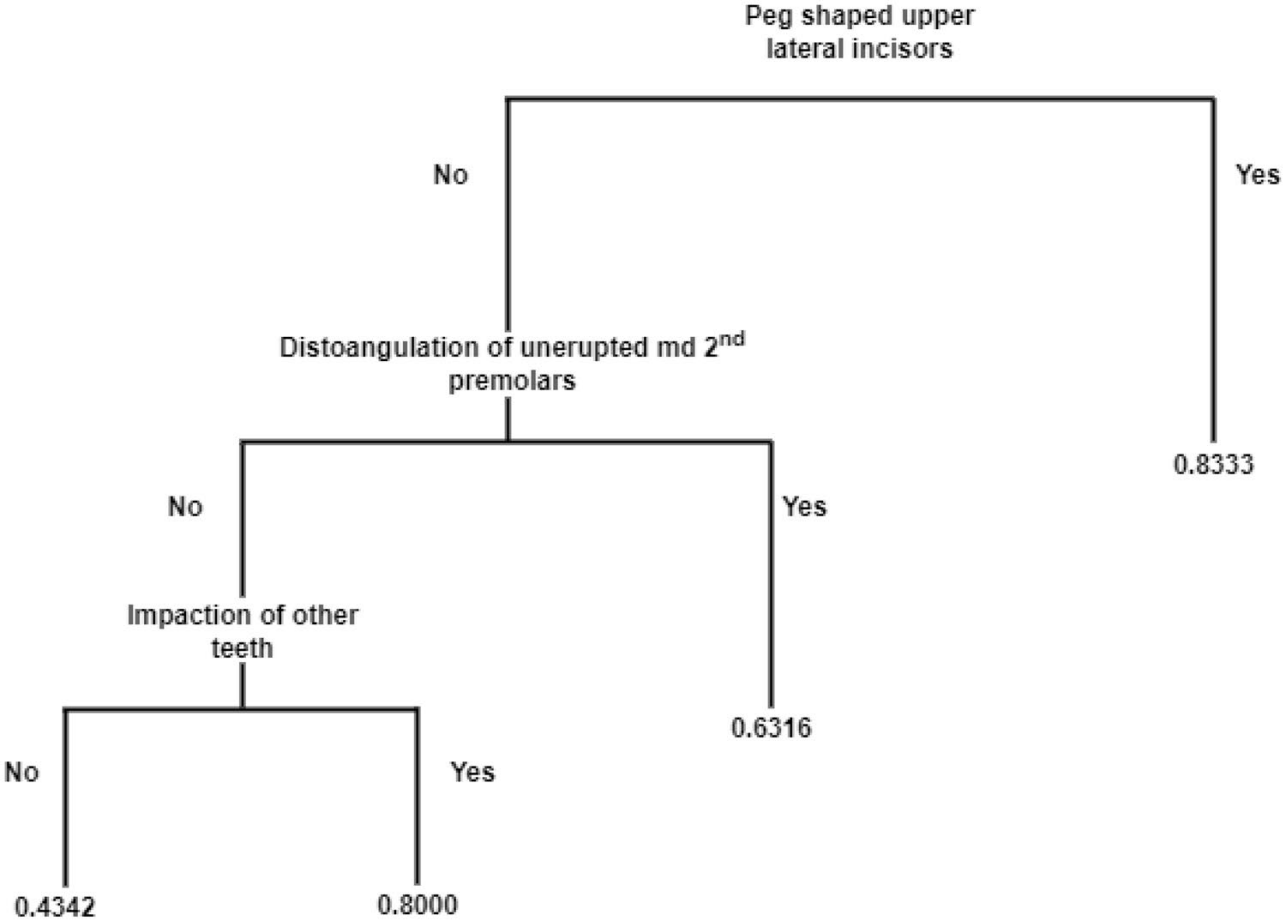
Classification tree

If we consider only the “No” category of the peg-shaped upper lateral incisors, it seems that there is an effect of the distal displacement of the unerupted mandibular second premolars. Specifically, the effect of “Yes” category of the distal displacement of the unerupted mandibular second premolars creates a subcategory of being classified in the impacted group with an increased probability of 63.16%.

In the more complex cases where peg-shaped upper lateral incisors and distal displacement of the unerupted mandibular second premolars are both “No”, the presence of “Yes” in the variable impaction of other teeth creates a subcategory of increased probability (80%) of being classified in the impacted group.

Notably, the second statistically significant variable found by traditional analysis, the infraocclusion of primary molars, does not appear in the tree.

## Discussion

The present study focused on the possible associations between dental anomalies and maxillary canine impaction group (MCIG) in a sample of Caucasian subjects, when compared with a control group (CG) of subjects without impaction. No association was demonstrated between dens invaginatus, dens evaginatus, taurodontism, germination and MCIG.

A statistically significant association between peg-shaped lateral incisors and infraoccluded deciduous molars and MCI was found. MCI group exhibited significantly greater prevalence of peg-shaped lateral incisors (15.5%) compared to the control group (3.1%) as previously reported (Peck et al. 1996; Sacerdoti and Baccetti 2004; Anic-Milosevic et al. 2009; Shalish et al. 2010; Singler et al. 2011; Mercuri et al. 2013; Garib et al. 2015).

Primary molar infraocclusion have been reported as a risk factor for impaction of maxillary canines (Shalish et al. 2010; Garib et al. 2016). The prevalence of primary molar infraocclusion in the MCI group found 5.2%.

Singler, Baccetti and McNamara (2011) showed that small lateral incisors, infraocclusion of primary molars, and distally displaced erupting mandibular second molars are significantly associated with maxillary impacted canines compared with a control group.

Although in this study is not found a statistically significant difference for distal displacement of unerupted mandibular second premolar, there is a greater prevalence rate of 12.4% in the impaction group compared to the control group that the corresponding percentage is 7.2%. Baccetti and wo-workers (2010) have demonstrated a significant relationship between distal displacement of unerupted mandibular second premolar and displaced canines and reported that the incidence of distal displacement of unerupted mandibular second premolar can be a developmental risk indicator for displaced maxillary canines. Also, Garib and wo-workers (2016) reported the above dental anomaly as an early risk marker for displaced canines.

This study did not demonstrate any association between mandibular second premolar agenesis and the MCIG. This finding agrees with a recent study conducted by Lagana and co-workers (2018) that showed that the mandibular second premolar or other types of agenesis except for agenesis of maxillary lateral incisors did not show any significant association with maxillary displaced canines. Also agrees with the retrospective cohort study of Garib and co-workers (2016) that the above association was not significant. In contrary, previous cross-sectional studies have showed that mandibular second premolar agenesis associated with maxillary displaced canines (Peck et al. 1996; Bacccetti 1998; Garib et al. 2009).

Some authors related the impaction of maxillary canines to the agenesis of maxillary lateral incisors ( Al-Nimiri 2005; Al-Nimiri 2011; Sacerdoti and Baccetti 2004). In this study, no significant associations were found between agenesis of maxillary lateral incisors or others agenesis except third molars and the MCIG. Peck and co-workers (1996), Mercuri and co-workers (2013) and Garib and co-workers (2016) also showed that agenesis of maxillary lateral incisors was not associated with MCIG.

An attempt to find relations between the different variable and the impacted/control group with a classification tree was made. Most significant variable for the classification of impacted/control group was found the peg-shaped upper lateral incisor. The presence of a peg-shaped upper lateral incisor arises the probability of an impacted canine to 83.3%. In absence of a peg-shaped upper lateral incisors, the presence of a distal displaced unerupted mandibular second premolar, in the same patient, increases the probability of maxillary canine impaction to 63.16%. In the more complex cases where peg-shaped upper lateral incisors and distal displacement of the unerupted mandibular second premolars are not exist, the presence of other tooth impaction increases the probability of maxillary canine impaction to 80%. This is a new knowledge of probabilities of maxillary impaction associated with the presence of different dental anomalies and should make clinicians feel significantly more powerful in their diagnostic capability.

A limitation of this retrospective study is that potential maxillary impaction was assessed using radiographic criteria only. This may lead to false-positive diagnosis. Since the false-positive rate has been found as low as 4.22%, seems not to compromise the study results (Lindauer et al. 1992). To ensure more accurate diagnosis, the presence of a palatal bulge, the amount of space for eruption, delayed eruption of the permanent canine or prolonged retention of the primary canine, position of the adjacent teeth, the contour of the bone, the mobility of teeth could be considered through clinical evaluation.

The results of this study show that peg-shaped upper lateral incisors, infraoccluded primary molars, distal displaced unerupted mandibular second premolar and impaction of any other teeth can be considered early risk indicators for maxillary canine impaction, because such dental anomalies manifest before maxillary canine impaction becomes apparent.

Maxillary canines’ eruption disturbances are common clinical problems affecting the developing permanent dentition, causing major complications such as root resorption of an adjacent tooth (Ericson and Kurol 2000; Hadler-Olsen et al. 2015, Ristaniemi et al 2022). Furthermore, late treatment for impacted canines is usually a long term, expensive procedure (Bazargani et al. 2013). Thus, early recognition of these dental anomalies during early mixed dentition is crucial and can increase early diagnosis of maxillary canine impaction and intervention.

## Conclusion

Peg-shaped maxillary lateral incisors, infraocclusion of primary molars, distal displaced unerupted mandibular second premolar and impaction of any other teeth are shown to be the major early developmental risk indicators for maxillary canine impaction. Additionally, distal displaced unerupted mandibular second premolar and the impaction of any other teeth have been identifying valuable as early risk indicators, by increasing the probability of maxillary canine impaction to 63.16% and 80%, respectively. Patients with these dental anomalies are candidates for future interceptive treatment for canine eruption.
